# Protocols in the therapeutic use of medical ozone: a matter of debate

**DOI:** 10.4103/mgr.mgr_15_23

**Published:** 2024-03-28

**Authors:** Salvatore Chirumbolo, Luigi Valdenassi, Sergio Pandolfi, Francesco Vaiano, Giovanni Ricevuti, Umberto Tirelli, Vincenzo Simonetti, Marianno Franzini

**Affiliations:** Department of Engineering for the Innovation Medicine (DIMI), University of Verona, Verona, Italy; Italian Scientific Society of Oxygen Ozone Therapy, Bergamo, Italy; Tirelli Medical Group, Pordenone, Italy; Department of Drug Science, University of Pavia, Italy

Dear Editor,

We read the recent article in this journal by Dengiz et al.,^1^ in which the authors used ozone via a nebulization approach as an adjuvant for the treatment of coronavirus disease 2019 (COVID-19) pneumonia. In their prospective randomized trial, Dengiz and colleagues performed an ozone inhalation treatment in a cohort of 15 patients undergoing standard COVID-19 pharmacological therapy and compared them with another 15 patients treated with standard COVID-19 therapy alone (controls).^1^ The ozone group underwent three sessions of 0.2 ppm ozone (O_3_), inhaled via a cold water vapor with 0.2 ppm O_3_ in olive oil for 10 minutes each at 5-minute intervals for 5 days.^1^ The authors reported improvements in hospital length of stay, inflammatory biomarker levels (C-reactive protein), and chest CT severity scores.^1^

The use of medical ozone, usually in a balanced gaseous mixture with oxygen, to treat inflammatory diseases such as COVID-19, relies on at least two fundamental approaches, i.e., autohemotherapy and rectal insufflation, whereas the study by Dengiz et al.,^1^ with its proposal to use ozone inhalation, represents an unexpected novelty.^2^

Current scientific literature is particularly critical with the use of O_3_ as a medical treatment, due to ozone toxicity. As a matter of fact, the American Conference of Governmental Industrial Hygienists established that the threshold limit value-time-weighted average for ozone recommended an upper limit of 0.1 ppm O_3_, to be not exceeded at any time, therefore we wondered how Dengiz et al.,^1^ decided to overcome this limit and even use a doubled dosage of O_3_ for their inhalation treatment.^1^,^3^

Previous evidence has reported a deleterious effect of inhaled ozone on pulmonary surfactant proteins SP-B and SP-C even at an exposure of 0.08 ppm O_3_ for only 2 hours,^4^ while COVID-19, in its severe form, can promote an IgA autoantibody-mediated damage towards SP-B and SP-C, resulting in poor oxygenation due to alveolar collapse.^5^ The activity of SP-B and SP-C is therefore crucial for the proper lung physiology.

Furthermore, lipids in the surfactant are of utmost importance. They provide an immune protection against microbial insults^6^,^7^ and their chemical modification via ozone-mediated peroxidation, might change the pulmonary immune microenvironment.^8^ Lungs have a high proportion of polyunsaturated fatty acid, particularly ω3-polyunsaturated fatty acid and other unsaturated fatty acids and ozone has a high capability to directly generate gross amounts of lipoperoxides and 4-hydroxy-2,3-trans-nonenale, which are particularly toxic at high doses.^8^ Therefore, on the basis of this criticism, we wonder how Dengiz et al.^1^ reported apparently encouraging evidence.

Actually, there are some bewildering issues in the manuscript that caused us to be perplexed. For example, the authors clearly reported in the Introduction that inhaled ozone is toxic and that the usual way to employ O_3_ in the medical treatment is O_3_ autohemotherapy, so, it is not clear why the authors used ozone inhalation.^1^ A significant percentage of patients (43%) suffered from chronic obstructive pulmonary disease, but the authors did not include these patients in the exclusion criteria, to avoid statistical confounding with COVID-19 pneumonia. The patients were therefore poorly described. The authors did not categorize their patients into different clinical phenotypes based on different degrees of COVID-19 severity, and, in this context, the inclusion criteria might have included patients without any COVID-19 symptoms.^1^ Moreover, no statistical evaluation of the sample size effect was performed. The evaluation of C-reactive protein is, again, particularly puzzling. While the cumulative C-reactive protein levels gave mean values for the O_3_-treated *vs*. control patients showing no statistical significance, the authors collected and separated different C-reactive protein values from the cohorts and subjected these single values to statistics, without detailing how. Fundamentally, the whole set of results between the two separate groups gave, at most, not significant data. The question we would like to put here is: why using an adjuvant therapy approach via the inhalation route rather than autologous blood?

Because of the different density of O_3_ in air respect to water (for example blood plasma), 0.2 ppm in air (or a complete lipophilic medium) corresponds to 428 mg/L O_3_, while 0.2 ppm corresponds to 0.2 mg/L into water. Actually, the amount of ozone (in mg/L) via inhalation is higher than the same dose in water. The Italian Scientific Society of Oxygen Ozone Therapy recommends major ozone autohemotherapy, even combined with ozone rectal insufflation, for the treatment of COVID-19 patients.^9^,^10^ The recommendation to use blood or rectal mucosa is to avoid a massive production of lipoperoxides, which on the contrary is achieved when O_3_ is inhaled, due to the particular physiology and biochemistry of the lungs.^11^ O_3_ acts via a mito-hormetic mechanism, which is a mechanism that occurs aside from the toxicological range of O_3_,^12^,^13^ and in this sense circulating blood is a more complex milieu where O_3_ is rapidly quenched into a panoply of intermediates, at low concentrations, and rather than into a cascade of lipoperoxides and hydroperoxides, due to the direct chemical reaction of ozone with lipids in the surfactant. We would be grateful if the authors would clarify these points in a rebuttal.
